# Unraveling Arbuscular Mycorrhiza-Induced Changes in Plant Primary and Secondary Metabolome

**DOI:** 10.3390/metabo10080335

**Published:** 2020-08-18

**Authors:** Sukhmanpreet Kaur, Vidya Suseela

**Affiliations:** Department of Plant and Environmental Sciences, Clemson University, Clemson, SC 29634, USA; sukhmak@clemson.edu

**Keywords:** arbuscular mycorrhizal fungi, metabolomics, plant–microbe interactions, rhizosphere, secondary metabolites, symbiotic association

## Abstract

Arbuscular mycorrhizal fungi (AMF) is among the most ubiquitous plant mutualists that enhance plant growth and yield by facilitating the uptake of phosphorus and water. The countless interactions that occur in the rhizosphere between plants and its AMF symbionts are mediated through the plant and fungal metabolites that ensure partner recognition, colonization, and establishment of the symbiotic association. The colonization and establishment of AMF reprogram the metabolic pathways of plants, resulting in changes in the primary and secondary metabolites, which is the focus of this review. During initial colonization, plant–AMF interaction is facilitated through the regulation of signaling and carotenoid pathways. After the establishment, the AMF symbiotic association influences the primary metabolism of the plant, thus facilitating the sharing of photosynthates with the AMF. The carbon supply to AMF leads to the transport of a significant amount of sugars to the roots, and also alters the tricarboxylic acid cycle. Apart from the nutrient exchange, the AMF imparts abiotic stress tolerance in host plants by increasing the abundance of several primary metabolites. Although AMF initially suppresses the defense response of the host, it later primes the host for better defense against biotic and abiotic stresses by reprogramming the biosynthesis of secondary metabolites. Additionally, the influence of AMF on signaling pathways translates to enhanced phytochemical content through the upregulation of the phenylpropanoid pathway, which improves the quality of the plant products. These phytometabolome changes induced by plant–AMF interaction depends on the identity of both plant and AMF species, which could contribute to the differential outcome of this symbiotic association. A better understanding of the phytochemical landscape shaped by plant–AMF interactions would enable us to harness this symbiotic association to enhance plant performance, particularly under non-optimal growing conditions.

## 1. Introduction

Plant–microbe interactions are ubiquitous and are indispensable for the health of both plants and soil. In belowground ecosystems, much of these interactions occur in the rhizosphere, which is inhabited by 10–100-fold more microbiome than the bulk soil due to the carbon (C) input from the plant [1]. Plant–microbe interactions often result in a positive outcome when plants associate with beneficial microorganisms such as mycorrhizal fungi, rhizobia, endophytes, and plant growth-promoting bacteria. The beneficial microbes enhance plant health through the acquisition of nutrients [2] and/or by enhancing the tolerance to abiotic [3] and biotic stresses [4] in return for the photosynthates [5]. A myriad of metabolites often mediates these interactions between plants and beneficial microbes as they facilitate partner recognition, colonization success, and hence the benefits gained by both partners. The microbial genome is affected by plant metabolites, and conversely, the plant genome is influenced by microbial metabolites; thus, the plant–microbe association induces changes in the transcriptome, proteome, and finally, the metabolome of the plant and microbes [6]. In this review, we focus on the changes in the primary and secondary metabolites in plants as influenced by the symbiotic association with arbuscular mycorrhizal fungi (AMF). We chose AMF, as it is among the most ubiquitous plant mutualists that enhance plant growth and yield mainly through the uptake of phosphorus (P). Phosphorus is sparingly available in soil due to complexation with iron and aluminum in acid soils and calcium in calcareous soils [7]. To increase the availability of P, plants have developed specific strategies that include the modification of root morphology, increase the rooting volume [8], the exudation of organic anions that releases P from metal complexes [9] and enzyme phosphatases that recycles the organic forms of P [10]. Another effective P acquisition strategy is the symbiotic association with AMF that allows the plants to forage a greater soil volume [11]. 

AMF are root obligate biotrophs belonging to the Glomeromycota phylum of fungi [11], forming symbiotic association with 70–80% of plants [12]. The symbiosis between plants and AMF is the most ancient plant-mutualistic association that has evolved over 400 million years ago [13,14]. The benefits of this mutualistic association to plants are multiple and varied, with the most important being the transfer of nutrients, mainly P [15,16], some extent of nitrogen (N), and other micronutrients such as copper and zinc due to their extensive hyphal network [17,18,19]. Higher procurement of nutrients by AMF is credited to their thin fungal hyphae that explore a larger volume of soil and could reach areas where the roots cannot access [20]. In addition to increasing the rooting volume, AMF can acquire P from less bioavailable P-minerals through chelation, acidification, and enzyme activity [21,22]. AMF symbiosis also increases tolerance to drought and salt stress and pests and diseases [23,24,25,26,27,28,29]. Apart from the acquisition of mineral nutrition, AMF also provides additional benefits by increasing the quality of the plant products [30,31] and contributes significantly to the accrual of soil organic matter through better soil aggregation and hyphal input [32]. Being the most widespread symbiotic association in nature, there is a high potential to utilize AMF symbiosis in agriculture, particularly in low input farming and sustainable agriculture [33]. Such an approach would reduce the need for P fertilizers, thus saving farming cost, and would further limit environmental pollution caused by the export of excess P to water bodies [34].

Unlike legume-rhizobia associations that exhibit high host specificity, the plant–AMF associations are less structured. The same plant can associate with different species of AMF and the same AMF species can associate with multiple plant species, i.e., they lack specificity in association. However, not all plant–AMF associations are mutually beneficial, and the productivity of the host plant (i.e., the outcome of the association) is highly specific to the identity of the AMF symbiont. For example, the AMF species *Glomus macrocarpum* caused a detrimental effect on tobacco plants [35]. However, other plant species such as *Sorghum bicolor* increased biomass and nutrient uptake when associated with *G. macrocarpum* [36]. Similarly, different AMF species stimulated different growth responses in *Sorghum bicolor* [37]. This differential response in the outcome of the plant–AMF association could be due to differences in both plant and AMF genotypes and environmental conditions [35]. The fungal characteristics that may contribute to the specificity in the outcome include differences in the functional traits [38], physiology [11], genetic variability [39], and molecular features such as signaling molecules and fungal effectors [40]. In addition, the AMF species can vary in their P uptake efficiencies [41], which may also contribute to the specificity in the outcome since the plant P uptake pathway is suppressed in the plant–AMF association [42]. Growth depressions in plants when associated with AMF may also arise due to the less control of the plant over the carbon allocation to AMF as plant may consider AMF colonization as a signal for providing carbon even in the absence of P benefits from the AMF [43]. Rhizosphere microbiome can also contribute to differences in the outcome of plant–AMF symbiosis as other soil microbes have a synergistic or antagonistic effect on root colonization by AMF [44,45].

The differential ability of plant species and AMF species to modulate plant metabolites could also contribute to the specificity in the outcome of plant–AMF association. Various metabolites influence the plant–AMF symbiotic association, starting from the pre-symbiotic stage to the formation and establishment of a functional symbiotic association. During the pre-symbiotic stage, the plants and the beneficial microbial partners exchange several chemical signals that result in the initiation of the symbiotic association. Under P-deficient conditions, several plant metabolites are exuded in the rhizosphere to ensure germination and growth of the AMF, whose concentration diminishes with increasing plant benefits from AMF colonization [46]. For example, the strigolactones are important signaling compounds from plants that affect the germination of the AMF spores and leads to the initiation of the plant–AMF symbiosis [47,48]. Apart from strigolactones, plant secondary metabolites such as flavonoids also act as chemical signals during the pre-symbiotic stage. Flavonoids are important for hyphal growth and their effect varies with their chemical composition [49]. Along with the plant metabolites, the AMF also produces certain chemical signals collectively termed as “myc-factors” [11] that initiate AMF colonization in plants. The lipochitooligosaccharides (LCOs) released by AMF act as signals that induce the symbiotic-specific response in host plants, which in turn helps in the development of the symbiosis [50]. The plant–AMF signaling has been elegantly elucidated by others [51,52] and is not the focus of this review. Here, we attempt to elucidate the changes in the primary and secondary metabolites in plants that define plant–AMF associations. 

**Figure 1:**
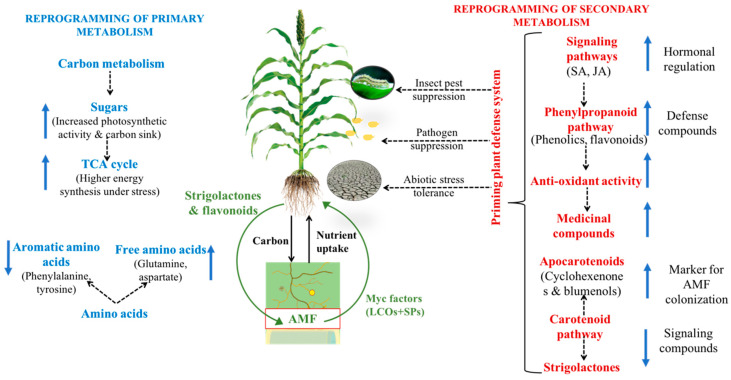
Schematic representation of the potential pathways through which arbuscular mycorrhizal fungi (AMF) reprograms the plant metabolome. Plant–AMF symbiosis reprograms the primary and secondary metabolites in plants. Reprogramming of secondary metabolites autoregulates the AMF colonization in the plant by modulating the production of signaling compounds. Interaction of plants with AMF primes plant defense via changes in the secondary metabolic pathways, which increase plant tolerance to biotic and abiotic stresses. Key: LCOs—lipochitooligosaccharides; SPs—secreted proteins; SA—salicylic acid, JA—jasmonic acid.

Apart from the chemicals produced exogenously to recognize partners and to establish a functional symbiosis, this mutualistic association also result in the reprogramming of the primary and secondary metabolic pathways of plants (Figure 1; [25,53,54]). The change in the plant metabolic pathways could be potentially mediated by the AMF, plants, environment, and their interactions. The change in plant metabolome due to the symbiotic association with AMF can vary with different plant species or colonization with different AMF species.

## 2. Primary Metabolites

In the plant–AMF association, plants transfer nearly 4–20% of the photosynthetically fixed carbon to the AMF [25] in the form of sugars [55] and lipids [56]. The carbon drain from the plant by AMF leads to a higher photosynthetic activity [57] as well as creates a carbon sink in the roots [55]. Thus, the carbon metabolism is often elevated in plants inoculated with AMF compared to the non-inoculated control plants. Moreover, these variations are often specific to AMF strains and plant species [58]. AMF-mediated changes in the root metabolites can also affect the metabolites of aboveground organs either due to signaling or transportation [59,60]. Previous studies have revealed that plants associated with AMF differ considerably from non-inoculated plants in the primary metabolites such as sugars, organic acids, and amino acids [61,62,63,64].

### 2.1. Sugars

Sugar forms an important regulator in the plant–AMF symbiotic association [56,65] as it is among the main sources of carbon supply from the plants to the AMF. Hexoses such as glucose and fructose are easily assimilated by the AMF structures in the roots [66]. A higher concentration of hexoses was observed in the roots at the beginning of the AMF colonization (26–29 days after germination). However, at harvest (40 days after germination), the non-mycorrhizal roots exhibited higher sugars compared to mycorrhizal roots [67]. Maize plants had a higher concentration of sugars in the leaves when associated with AMF [68]. As the plant–AMF interactions are highly functional under low nutrient availability [11], higher sugar content is observed in mycorrhizal plants under P deficiency (Table 1). For example, cebil seedlings in association with an AMF species mixture showed a higher accumulation of soluble carbohydrates and proteins under P-deficient conditions. However, this response was not observed in the seedlings supplied with high P, where there was less colonization by AMF, indicating the potential of AMF to reprogram sugar metabolism [69]. The hexoses assimilated by AMF can be further transformed into trehalose and glycogen in the intra-radical mycelium of AMF [55]. Trehalose is considered as a fungal-specific sugar due to its presence only in the mycorrhizal plants [70]. The concentration of trehalose increased with mycorrhizal colonization [71], and its accumulation in mycorrhizal plants can protect the plants against abiotic stresses [72].

### 2.2. Organic Acids

In addition to sugars, AMF also impacts the tricarboxylic acid (TCA) cycle [64]. The TCA cycle and its intermediaries involving citric acid, malic acid, fumaric acid and succinic acid are important for cellular respiration and adenosine triphosphate (ATP) synthesis [73]. This energy synthesis helps the plant in its development as well as tolerating adverse stress conditions [74]. For example, during the later stages of mycorrhizal development, higher accumulation of organic acids was observed in the leaves of pea [75]. However, a decrease in organic acids of the TCA cycle was recorded in dicots along with species-specific changes in other metabolites in the above-ground organs of plants as AMF affects the plants’ central catabolism due to carbon utilization. In contrast, an increase in organic acids in the monocot (*Poa annua*) was observed (Table 1; [76]). Some of the substrates of the TCA cycle such as aconitic acid and fumaric acid diminished in mycorrhizal roots of *Medicago truncatula*, implying a mycorrhiza induced stimulation of mitochondrial and plastidial metabolism [70]. Moreover, plants release organic acids into the soil to solubilize the insoluble form of P [77,78]. For example, a higher concentration of citric acid was observed in the soil solution of *Allium Cepa* colonized with *Gigaspora margarita* compared to the control plants [79]. AMF species can also vary their ability to release organic acids into the soil [80]. Fungal extra-radical hyphae release organic acids into the soil which helps in breaking the complex forms of minerals and therefore could increase their absorption in the plants [81].

### 2.3. Amino Acids

Amino acids, the building blocks of proteins, and enzymes, also act as signaling molecules and regulates environmental stress in plants [82]. The amino acids can be directly taken by the AMF from the soil [62] and they can also be synthesized by AMF spores using N from the soil [83]. The amino acid uptake from the soil was higher in the arbuscular mycorrhizal plants compared to the non-mycorrhizal plants [62]. Previous studies have reported increased, decreased or no variation of amino acid content in plants due to AMF colonization (Table 1; [84,85]). AMF colonization decreased the content of phenylalanine, the precursor of phenylpropanoids. Amino acids such as tryptophan, tyrosine, phenylalanine, alanine, and leucine decreased, while the abundance of amino acid-derived secondary metabolites increased in the mycorrhizal roots of tomato [64]. Concomitantly, amino acids such as aspartic, asparagine, glutamic, and pyroglutamic acids that are less related to the production of secondary compounds increased in AM roots [70]. Amino acids such as glutamic acid, histidine, and cysteine accumulated in tomato plants associated with AMF [86]. Furthermore, there was no variation in the amino acid content of mycorrhizal and non-mycorrhizal roots of tobacco plants [87]. 

Similar to the roots, amino acids were higher in the leaves of different plant species inoculated with AMF [75,88] compared to the non-inoculated plants. However, a reduction in the amino acids was observed in willow leaves [89] and *Lotus japonicus* associated with AMF [84]. Apart from roots and leaves, the amino acid content in fruits also increased with mycorrhization. For example, in tomato plants inoculation with *Glomus mosseae* increased several amino acids in fruits, of which glutamine and asparagine concentrations were the most responsive [90]. Aromatic amino acids are important in grapes as they are essential for the aroma of wines and these amino acids increased in grapes when colonized by AMF species compared to the non-inoculated plants [91]. Amino acids showed variable accumulation in different studies which could be a function of the plant species or fungal genotype as well as the environmental conditions (Table 1). 

### 2.4. Effect of AMF on Primary Metabolites under Biotic/Abiotic Stresses

Plants subjected to abiotic stress increased the content of sugars particularly in association with AMF. Under water-stressed conditions, both sugars and lipids were upregulated in AMF plants, which was consistent with the biomass increase in the plants [92]. Similarly, under drought conditions, maize inoculated with AMF had less reduction in sugar content compared to the non-inoculated plants [93]. Although a higher abundance of reducing sugars was observed in the leaves of mycorrhizal maize plants without salinity stress, comparatively higher reducing sugars were observed in mycorrhizal plants compared to non-mycorrhizal plants at different salinity levels [68]. Increased soluble sugars by mycorrhizae rendered resistance against salt stress in maize plants [61]. Several studies have found a similar accumulation of sugars in plants colonized by AMF under different abiotic stress conditions such as drought, salinity, cold stress, heavy metals, and nutrient deficiencies [81,94,95,96,97,98,99,100,101,102,103]. The accumulation of sugars could vary with AMF species. For example, trifoliate orange seedlings under drought conditions accumulated higher concentrations of sucrose, glucose, and fructose in the leaves of the plants inoculated with *Paraglomus occultum* compared to *Funneliformis mosseae* [29]. Moreover, under stress conditions, there could be a differential accumulation of sugars in roots and leaves (Table 1). For example, under temperature stress, soluble sugars accumulated in roots of AMF plants but the soluble sugar content in leaves did not vary between AMF and non-inoculated plants [104]. 

An increase in organic acids was observed in *Puccinellia tenuiflora* inoculated with AMF, as part of the mechanism of toleration against alkali stress [105]. Similarly, monocots such as *Poa annua* [76] and maize associated with AMF had higher organic acid content under salt stress [68]. A higher concentration of malic acid was observed in the root exudates of *Citrullus lanatus* (watermelon) colonized with *Glomus mosseae*. However, other organic acids increased with *Fusarium oxysporum* f. sp. niveum infection [106]. 

Under drought conditions, trifoliate orange plants inoculated with AMF plants had a lower accumulation of proline than the non-AMF plants indicating that AMF plants experienced less stress under drought [29]. In soybean, under drought stress, the roots of AMF plants accumulated 14% more proline, whereas the shoots accumulated 39% less proline compared to the non-inoculated plants [107]. Interestingly, another study with maize showed a reduction in amino acids in the leaves of mycorrhizal plants under different salinity levels [68]. Furthermore, under alkaline stress conditions, amino acids such as glutamine that scavenge reactive oxygen species (ROS) also increased in the AMF-inoculated seedlings of *Puccinellia tenuiflora* [105]. More importantly, in cocoa plants, AMF inoculation reduced the susceptibility to *Phytophthora megakarya* along with higher soluble content of amino acids in leaves [108].

Overall, the sugars, organic acids, and amino acids are differentially regulated in various plant species by different AMF species (Table 1). They are mostly elevated or decreased, to the best benefit of the plant and also to reduce the costs of the resource under biotic and abiotic stresses. Moreover, AMF also showed the capacity to upregulate or downregulate plant metabolism based on plant growth conditions. The changes in primary metabolites due to AMF provide protection from environmental stress and improves the quality of the plant products. Most of these benefits to plants may also depend on the AMF demand on plant carbon, which would also vary with different AMF species thus affecting the specificity of the outcome of plant–AMF interaction. 

**Table 1:** Summary of primary metabolites modulated by AMF in different studies.

Primary Metabolites	Plant Parts	AMF Species	Plant Species	Enviromental Condition	Increase/Decrease	Reference
**Sugars**						
Fructose	Roots	*Glomus versiforme*	*Poncirus trifoliata*	Well-watered	Increase	[109]
Glucose	Roots	*Glomus versiforme*	*Poncirus trifoliata*	Drought	Increase	[109]
Kestose	Leaves, flowers	*Glomus mossae*	*Lotus japonicus*	-	Increase	[84]
Soluble sugars	Root + shoot	*Glomus mossae*	Maize	Salt stress	Increase	[61]
Soluble sugars	Leaves	*Glomus mossae*	Maize	Salt stress	Increase	[68]
Sucrose	Roots + leaves	*Glomus versiforme*	*Poncirus trifoliata*	Drought	Increase	[109]
Total sugars	Root	*Glomus intraradices*	Maize	Drought	Increase	[93]
Total sugars	Leaves	*Glomus intraradices*	Maize	Drought	Increase in drought sensitive and decrease in drought resistant variety	[93]
Trehalose	Roots	*Glomus intraradices*	*Medicago truncatula*	-	Increase	[70]
**Sugar alcohols**						
Myo-inositol	Leaves, flowers	*Glomus mossae*	*Lotus japonicus*	-	Increase	[84]
Pinitol	Roots	*Funneliformis mossae*	*Triticum durum*	Water stress	Decrease	[92]
Xilitol	Roots	* AMF mix and natural AMF inoculum	*Triticum durum*	N stress, P rich	Increase	[110]
Xylitol	Leaves, flowers	*Glomus mossae*	*Lotus japonicus*	-	Increase	[84]
**Organic acids**						
2-methyl-malic acid	Leaves, flowers	*Glomus mossae*	*Lotus japonicus*	-	Decrease	[84]
Acetic acid	Leaves	*Glomus mossae*	Maize	Salt stress	Increase	[68]
Citric acid	Leaves, flowers	*Glomus mossae*	*Lotus japonicus*	-	Decrease	[84]
Citric acid	Leaves	*Rhizophagus irregularis*	** Monocot and dicots	-	Decrease in dicots and increase in monocot	[76]
Formic acid	Leaves	*Glomus mossae*	Maize	Salt stress	Decrease at highest salt concentration	[68]
Fumaric acid	Leaves	*Glomus mossae*	Maize	Salt stress	Increase	[68]
Fumaric acid	Leaves	*Rhizophagus irregularis*	** Monocot and dicots	-	Decrease in dicots and increase in monocot	[76]
Isocitric acid	Leaves	*Rhizophagus irregularis*	** Monocot and dicots	-	Partly Decrease in dicots and increase in monocot	[76]
Malic acid	Leaves, flowers	*Glomus mossae*	*Lotus japonicus*	-	Decrease	[84]
Malic acid	Leaves	*Glomus mossae*	Maize	Salt stress	Increase	[68]
Malic acid	Leaves	*Rhizophagus irregularis*	** Monocot and dicots	-	Decrease in dicots and increase in monocot	[76]
Oxalic acid	Leaves	*Glomus mossae*	Maize	Salt stress	Increase	[68]
Succinic acid	Leaves, flowers	*Glomus mossae*	*Lotus japonicus*	-	Decrease	[84]
Succinic acid	Leaves	*Glomus mossae*	Maize	Salt stress	Decrease at highest salt concentration	[68]
Succinic acid	Leaves	*Rhizophagus irregularis*	** Monocot and dicots	-	Partly decrease in dicots and increase in monocot	[76]
**Amino acids**						
4-amino-butanoic acid	Leaves, flowers	*Glomus mossae*	*Lotus japonicus*	-	Decrease	[84]
Alanine	Leaves	*Rhizophagus irregularis*	** Monocot and dicots	-	Increase (mainly in dicots)	[76]
Alanine	Root	* AMF mix and natural AMF inoculum	*Triticum durum*	N stress, P rich	Decrease	[110]
Asparagine	Leaves, flowers	*Glomus mossae*	*Lotus japonicus*	-	Decrease	[84]
Asparagine	Roots	* AMF mix and natural AMF inoculum	*Triticum durum*	N stress, P rich	Decrease in AMF mix	[110]
Asparagine	Roots	*Glomus intraradices*	*Medicago truncatula*	-	Increase	[70]
Aspartic acid	Leaves, flowers	*Glomus mossae*	*Lotus japonicus*	-	Decrease	[84]
Aspartic acid	Leaves	*Rhizophagus irregularis*	** Monocot and dicots	-	Increase (mainly in dicots)	[76]
Aspartic acid	Roots	*Funneliformis mossae and Rhizophagus irregularis* *(different treatments)*	*Solanum lycopersicum*	Minimum P	Decrease	[64]
Aspartic acid	Roots	*Glomus intraradices*	*Medicago truncatula*	-	Increase	[70]
Glutamic acid	Leaves, flowers	*Glomus mossae*	*Lotus japonicus*	-	Decrease	[84]
Glutamic acid	Leaves	*Rhizophagus irregularis*	** Monocot and dicots	-	Increase (mainly in dicots)	[76]
Glutamic acid	Roots	*Funneliformis mossae and Rhizophagus irregularis* *(different treatments)*	*Solanum lycopersicum*	Minimum P	Increase	[64]
Glutamic acid	Roots	*Glomus intraradices*	*Medicago truncatula*	-	Increase	[70]
Glutamine	Roots	* AMF mix and natural AMF inoculum	*Triticum durum*	N stress, P rich	Decrease in AMF mix	[110]
Glycine	Leaves, flowers	*Glomus mossae*	*Lotus japonicus*	-	Decrease	[84]
Homoserine	Leaves	*Rhizophagus irregularis*	** Monocot and dicots	-	Increase (mainly in dicots)	[76]
Isoleucine	Leaves	*Rhizophagus irregularis*	** Monocot and dicots	-	Increase (mainly in dicots)	[76]
Leucine/Isoleucine	Roots	*Funneliformis mossae and Rhizophagus irregularis* *(different treatments)*	*Solanum lycopersicum*	Minimum P	Decrease	[64]
Ornithine	Leaves	*Rhizophagus irregularis*	** Monocot and dicots	-	Increase (mainly in dicots)	[76]
Phenylalanine	Leaves	*Rhizophagus irregularis*	** Monocot and dicots	-	Increase (mainly in dicots)	[76]
Phenylalanine	Roots	* AMF mix and natural AMF inoculum	*Triticum durum*	N stress, P rich	Decrease in AMF mix	[110]
Phenylalanine	Roots	*Funneliformis mossae and Rhizophagus irregularis (different treatments)*	*Solanum lycopersicum*	Minimum P	Decrease	[64]
Serine	Leaves	*Rhizophagus irregularis*	** Monocot and dicots	-	Increase (mainly in dicots)	[76]
Threonine	Leaves	*Rhizophagus irregularis*	** Monocot and dicots	-	Increase (mainly in dicots)	[76]
Tryptophan	Roots	*Funneliformis mossae and Rhizophagus irregularis* *(different treatments)*	*Solanum lycopersicum*	Minimum P	Decrease	[64]
Tyrosine	Roots	*Funneliformis mossae and Rhizophagus irregularis* *(different treatments)*	*Solanum lycopersicum*	Minimum P	Decrease	[64]
**Fatty acids**						
Fatty acids and their esters	Roots	* AMF mix and natural AMF inoculum	*Triticum durum*	N stress, P rich	Decrease in AMF mix	[110]
Octadecanoic acid	Leaves, flowers	*Glomus mossae*	*Lotus japonicus*	-	Decrease	[84]
Oleic acid	Roots	*Glomus intraradices*	*Medicago truncatula*	-	Increase	[70]
Palmitic acid	Roots	*Glomus intraradices*	*Medicago truncatula*	-	Increase	[70]
Palmitvaccenic acid	Roots	*Glomus intraradices*	*Medicago truncatula*	-	Increase	[70]
Vaccenic acid	Roots	*Glomus intraradices*	*Medicago truncatula*	-	Increase	[70]
**Proteins**	Leaves	*Gigaspora albida+ Acaulospora longula*	*Anadenanthera colubrina*	Minimal P	Increase	[69]
**Carbohydrates**						
3-propylphosphoenolpyruvate	Roots	*Funneliformis mossae*	*Triticum durum*	Water stress	Increase	[92]
Carbohydrates	Leaves	*Gigaspora albida+ Acaulospora longula*	*Anadenanthera colubrina*	Minimal phosphorusP	Increase	[69]
Glucose-1,6-bisphosphate	Roots	*Funneliformis mossae*	*Triticum durum*	Water stress	Increase	[92]
Mannosylfructose-phosphate	Roots	*Funneliformis mossae*	*Triticum durum*	Water stress	Increase	[92]

* AMF mix = *Scutellospora calospora*, *Acuolospora laevis*, *Glomus aggregatum*, *Rhizophagus irregulare*, *Funneliformis mossae*, *Glomus fasciculatum*, *Glomus deserticola*, and *Gigaspora margarita*. ** Monocot and dicots = *Plantago lanceolata*, *Plantago major*, *Veronica chamaedrys*, *Medicago truncatula* (Dicots) and *Poa annua* (Monocot).

## 3. Secondary Metabolites

Secondary metabolites play a critical role in plant defense against biotic and abiotic stresses [111]. Similar to primary metabolites, AMF also influences different secondary metabolites. In the plant–AMF symbiosis, the secondary metabolites mediate interactions between the two partners starting from the phase of recognition to colonization and further the establishment of AMF within the root tissues. Secondary metabolites are released by both plants and AMF in the pre-symbiotic stage of association, which act as signaling molecules in plant–AMF symbiotic interaction [112]. The host plant and AMF then engage in a coordinated molecular dialog that would suppress the defense response of the host plant [113] and later prime the host for better tolerance against biotic and abiotic stresses [114]. The AMF-induced plant protection against biotic and abiotic stresses is mediated through the production of several secondary metabolites (Table 2). The ability to modulate and suppress the plant defense system may vary with both plant and AMF genotypes and their interaction with the environment. Inoculation with AMF can improve plant health by enhancing the whole plant defense system known as the systemic acquired resistance (SAR). Furthermore, AMF can prime the plant to react faster to pathogen attack through induced systemic resistance ISR [115]. AMF also provides protection against many insect-pests where the AMF-induced plant protection was found to be more effective against phloem feeders or specialist chewing insects compared to generalist herbivores [4]. Other than their function in the plant defense system, some of the secondary metabolites enhanced by AMF in agricultural products [116] and medicinal plants [117] increase the quality of the products. Several secondary metabolite pathways are activated by AMF, such as the carotenoid, phenylpropanoid, and antioxidant pathways [118,119]. Compounds produced in these pathways are indicators of different functions in the plant–AMF symbiosis such as signaling, stress tolerance, nutrient uptake, and resistance against biotic and abiotic stresses (Figure 1).

### 3.1. Carotenoid Pathway

Carotenoids belong to the subfamily of terpenoids with 40 carbon atoms and 8 isoprene molecules in their structures [135]. The carotenoid pathway plays an important role in many plant processes such as photosynthesis, photoprotection, hormones synthesis, and release and signaling [136]. Some of the compounds derived from the carotenoid pathway are key regulators of AM symbiosis. Strigolactones, the important signaling molecules that initiate AMF symbiosis, are plant hormones that are synthesized in the carotenoid pathway [137]. Under low inorganic P availability, plants release strigolactones into the soil [138], which are perceived by the AMF as signaling molecules. The strigolactones trigger multiple responses in AMF such as germination of spores, hyphal elongation, and the formation of hyphopodia [47]. After colonization, the AMF in turn affects the carotenoid pathway by inducing the production of other apocarotenoids such as mycorradicin and cyclohexenone derivatives, which are important for establishing and maintaining AMF symbiosis [70,139]. For example, an accumulation of cyclohexenone derivatives was observed in barley with an increase in the mycorrhizal establishment [121]. Among the cyclohexonone derivatives, the most abundant are the mono-, di- and branched triglycosides of blumenols [125]. Blumenols which are produced in the roots were found to be further transported to the aboveground organs of the plants and are considered as shoot biomarkers for AMF colonization [59]. The content of blumenols could thus vary with plant organs where its content in the roots may not mirror that in the shoots as observed in ragwort [118]. The accumulation of these cyclohexenone derivatives was observed in different plant species with different AMF species, but their quantity varied across AMF species [123]. 

Moreover, the apocarotenoids are observed to be highly abundant in AMF roots of most of the plant species. These apocarotenoids may involve in the synthesis of signaling molecules, autoregulation of fungal colonization, and protection of plants against pathogens and ROS [140]. The carotenoid biosynthesis is activated in almost all the mycorrhizal plants and thus it can be assumed as a general characteristic of mycorrhizal plants [141]. Additionally, blumenin, a sesquiterpenoid cyclohexenone glycoside, accumulated in the roots of graminaceous plants with mycorrhizae but was not observed when the same plants were exposed to abiotic stress or pathogen or endophyte. Thus, the terpenoids metabolism can be specific to the AMF association with the plant [126]. Moreover, the release of volatile sesquiterpenes upon herbivore attack reduced in the mycorrhizal inoculated *Plantago lanceolata* plants compared to the non-inoculated plants, reflecting indirect upregulation of the defense system in the plant [142]. These findings suggest that carotenoid pathway has different applicability in AMF symbiosis such as signaling compounds during the pre-symbiotic stage; as markers of AMF colonization in both root and shoot after colonization; and as important compounds mediating the indirect priming of the defense system.

### 3.2. Phenylpropanoid Pathway

Phenylpropanoids form the largest group of compounds among secondary metabolites. They are biosynthesized from aromatic amino acid phenylalanine. They are further divided into five main groups including flavonoids, monolignols, phenolic acids, stilbenes, and coumarins [143]. They play a significant role in signaling, which is important for both plant development and defense [144]. The phenylpropanoid pathway undergoes significant reprogramming due to the AMF association. The AMF induces a change in both the abundance and composition of different plant secondary metabolites to ensure its colonization and establishment in plants. 

Flavonoids are an important group of compounds in the phenylpropanoid pathway. Flavonoids provide color, aroma, and taste to plants; inhibit the production of ROS due to biotic or abiotic stresses; and regulate the plant-symbiotic associations [145]. In plant–AMF interactions, the flavonoids play an essential role in initiating and restricting the AMF colonization. The flavonoid biosynthesis was upregulated in the mycorrhizal plant leaves [60]. Different flavonoids can have varying effects on the colonization of different AMF species [146]. The flavonoids, namely formononetin and ononin, have an essential role in the autoregulation of symbiotic association by limiting AMF colonization in the plant after it reaches a threshold [147]. Similarly, at the later stages of fungal colonization, the upregulation of some isoflavonoids such as daidzein, ononin, and malonylonin was observed in the mycorrhizal roots of *Medicago truncatula* [70]. A comparative study conducted between successfully colonized roots of *Medicago truncatula*, *Medicago sativa* and myc^-^ roots (incompletely colonized roots) of *Medicago sativa* suggested that 4′, 7-dihydroxyflavone flavonoid increased in mycorrhizal roots. However, it did not increase in myc^-^ roots, suggesting its important role in the growth of internal AMF structure [148]. Similarly, medicarpin levels were elevated during initial colonization, and subsequently reduced to negligible levels at the full development stage of the AMF. Interestingly, the decrease in medicarpin was not observed in myc^-^ roots indicating that the decrease in medicarpin is critical for the establishment and regulation of AMF colonization in Medicago [148]. More importantly, increased levels of some flavonoids in mycorrhizal roots such as formononetin malonyl glucoside (FGM), medicarpin malonyl glycoside (MGM), diadzein and coumestrol emphasizes on the improved ability of mycorrhizal plants to resist stresses [148]. Along with the above, flavonoids such as quercetin, which is known to have an important role as a phytochemical in grapes, are stimulated in mycorrhizal plants, and this increase was highly dependent on the grape varieties [129]. Although increasing soil P affected the accumulation of primary metabolites, the elevated P conditions did not affect the accumulation of flavonoids, tannins, and phenolics [69]. Similarly, in the leaves of *Medicago truncatula*, the abundance of flavonoids and anthocyanins increased in AMF plants even in the absence of any P uptake benefits to the plant [134]. These results suggest that secondary metabolites in the phenylpropanoid pathway are often reprogrammed by mycorrhizal colonization irrespective of the benefits provided by AMF including the increased uptake of nutrients.

Apart from flavonoids, other phenolic acids also act as signaling compounds in plant–microbial interactions [149] and as antioxidant compounds that protect the plants against many stress conditions. A higher abundance of phenolic acids also reflects a better quality of the plant products [150]. Increased abundance of phenolic compounds was observed in the leaves and roots of peanut inoculated with AMF [128]. Moreover, different phenolic acids vary in abundance in the same plant. For example, caffeic and chlorogenic acids decreased while ferulic acid increased in abundance in the mycorrhizal roots of tomato [151]. Furthermore, different AMF species increased the tolerance of date palm against bayoud disease by increasing the enzymatic activities of peroxidases and polyphenoloxidases, which further are associated with increasing phenolic compounds in the cell wall [152]. Therefore, a higher abundance of phenolic acids in mycorrhizal plants depicts the priming of the defense system of the plant, which further protects the plant from pathogens. Moreover, other phenolic acid derivatives showed altered concentration in mycorrhizal plants. The accumulation of hydroxycinnamate amides was observed in barley roots [128] and chicory roots along with the higher abundance of the derivatives of caffeic acids in AMF-inoculated plants [127]. Similarly, other compounds in the phenylpropanoid pathway such as coumarins and its hydroxyl forms had higher abundance in leaves of willow when colonized by AMF [89]. These coumarins can act as antimicrobial and antioxidant compounds. A higher abundance of these compounds in the roots could also affect the mycorrhizal colonization because of their antimicrobial properties, which explains the lower abundance of coumarins such as scopoletin and its glucoside scopolin in the roots of tobacco cultivar [87]. 

Most of the compounds in the phenylpropanoid pathway have antioxidant properties, which will protect the plants against ROS. Not only these antioxidants are beneficial for plants in stress conditions, but also these compounds provide medicinal properties to the plant [153]. AMF has been found to increase the accumulation of these compounds in plants [154]. This increase in the production of antioxidants could be partly contributed by the indirect benefits provided by AMF including nutrient (mainly P) uptake and partly due to the direct interaction with the plant, under a series of environmental conditions [155]. Under less water availability, mycorrhizae increased the antioxidant compounds in lettuce [120]. Interestingly, plants associated with AMF increased the synthesis of antioxidants such as rosmarinic and caffeic acids in sweet basil than the plants supplied with sufficient P. Thus, the increase in antioxidants in mycorrhizal plants cannot be attributed only to the P benefits. AMF also increased the medicinal value of purslane by increasing fatty acids, flavonoids, and antioxidant activity in the leaves of the plant under drought conditions [117]. More importantly, *Glomus mosseae* and *Glomus caledonium* increased the abundance of these antioxidant compounds, whereas *Glomus intraradices* did not elevate the synthesis of these compounds [148]. Furthermore, AMF species varied in enhancing the phenolic content and antioxidant activity in the two cultivars of globe artichoke, where *Clardeoglomus claroideum* 22W3 and *Funneliformis mosseae* IMA1 increased the antioxidant activity compared to the control, whereas other isolates of *Funneliformis mosseae*, *Rhizophagus irregularis*, and *Glomus sp.* did not affect the antioxidant activity [149]. Therefore, different AMF species can play a distinct role in increasing the production of these medicinal compounds [133]. This is further evident from one of the studies with mycorrhizal maize plants, where the leaves of the plants inoculated with *Acaulospora longula* accumulated higher flavonoids content compared to the control and the plants inoculated with *Claroideoglomus etunicatum* and *Dentiscutata heterogama* [156,157]. Differences in the accumulation of phenolics and flavonoids in various plant species by different AMF species points to the higher specificity in the outcome of the plant–AMF symbiotic association. However, the mechanisms underlying the higher specificity in the outcome of this association are not well explored.

### 3.3. Effect of AMF on Secondary Metabolites under Biotic/Abiotic Stresses

AMF provides resistance against many biotic and abiotic stresses [108,158,159,160,161,162,163,164,165,166,167,168]. Phenolic compounds are important secondary metabolites in disease suppression or defense mechanisms against other stress. Mycorrhizae enhance the phenolic content in plants, which could explain its role in protecting the plants against pathogens [128]. These phenolics are synthesized in higher quantity due to the upregulation of signaling pathways involving hydrogen peroxide, salicylic acid, and nitrogen oxide pathways in the colonized roots [169]. Similar upregulation of these pathways was observed in the mycorrhizal inoculated plants against citrus canker [161]. Furthermore, elevated flavonoid content corresponded with increased resistance against the tomato mosaic virus in the mycorrhizal tomato plants [170]. Other important low-molecular-weight antimicrobial compounds, namely phytoalexins, were accumulated in the roots when exposed to the pathogens in the presence of AMF. However, the absence of either AMF or pathogen did not influence the phytoalexins in the plant roots [131]. Under chilling stress, AMF increased the biomass of cucumber plants when exposed to low-temperature conditions than the plants without AMF under optimum and stressed conditions. Consistent with this biomass increase, AMF also raised the concentration of phenols, flavonoids, lignin, and many enzymes involved in the synthesis of secondary metabolites [119]. Despite a positive mycorrhizal growth response (MGR) by AMF under both drought and salinity conditions, only salinity stress seemed to affect the metabolite profile of tomato roots, because plants under water stress are more inclined to change the physiological processes, whereas salt stress can cause variations in antioxidant metabolites. The compounds in the mevalonate pathway, carotenoid pathway, oxylipins pathway, and lignans, which improves the stress tolerance, were accumulated in tomato plants inoculated with AMF compared to the control plants under salinity conditions [86]. Additionally, the phenylpropanoid pathway also upregulated under salt stress in the presence of AMF [171]. Similarly, water stress also affected the plant metabolome of wheat cultivars associated with AMF, as evident from the differential regulation of several metabolites in the secondary metabolite classes, including alkaloids, flavonoids, and terpenoids [92]. Drought results in oxidative stress and the accumulation of ROS, which causes damage to plants. In the presence of AMF, antioxidant compounds such as glutathione and 4-O-oxalyl-L-threonate reacted with ROS and reduced the oxidative stress in plants and hence caused less damage to plants due to drought. In addition, AMF also affected the accumulation of most of the glucosinolates [92] and cyanogenic glycoside [172]. Moreover, AMF modulated the polyamine metabolic pathway in trifoliate orange under drought stress where it increased the accumulation of putrescine and cadaverine, which enhanced drought tolerance by scavenging ROS, maintaining cell pH and ion homeostasis [173]. Interestingly, even under drought conditions, AMF enhanced the antioxidant activity of the medicinal plant, Purslane [117] and *Dracocephalum moldavica* [174]. Overall, AMF provides resistance against many biotic and abiotic stresses by increasing the abundance of several secondary metabolites (Table 2).

## 4. Plant Defense and Hormonal Regulation

Plants initially perceive AMF as putative pathogens, which results in the temporary activation of plant defense responses during the initial stages of colonization. The plants can regulate their defense responses through elicitor degradation, stimulating genes involved in regulating symbiotic association and hormonal regulation, including salicylic acid (SA) and jasmonic acid (JA) [175]. During the initial stage of colonization, AMF triggers the production of SA in plants. SA has a strong impact on the initial establishment of AMF, but not on the final root colonization by AMF [176,177]. As the higher content of SA reduces the colonization by AMF, this initial response is suppressed to ensure successful colonization by AMF. Moreover, while the upregulation of SA was observed during the early phase of mycorrhization, the upregulation of JA was observed during the late phase of mycorrhization when the salicylic acid decreases to ensure the establishment of AMF in the plant [25]. The up- and downregulation of these phytohormones explains the activity of AMF against the pathogens controlled by these hormones. Furthermore, AMF primes the plant defense system regulated by jasmonic acid. The upregulation of the jasmonic acid signaling pathway during plant–AMF interaction increases the production of defense compounds [25]. This priming of the defense system can vary with AMF genotypes [53]. The increase in JA enhanced the biosynthesis of secondary metabolites such as flavonoids and terpenoids [134]. A large number of fungal effectors are predicted to be released by mycorrhizal fungi, which could differentially affect the signaling pathways. Similarly, different AMF species are known to release different secreted proteins or fungal effectors, thus could affect the production of signaling hormones [40]. Precise regulation of different plant hormones is a critical factor in the successful establishment and hence the outcome of this symbiotic relationship [178]. 

## 5. Analytical Platforms for the Analysis of Plant Metabolome

**Figure 2:**
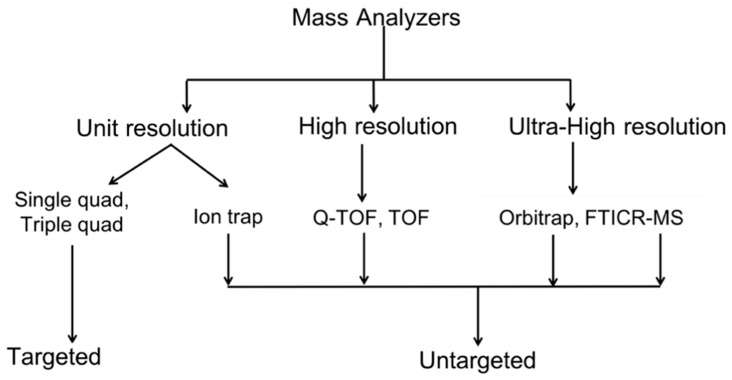
Different mass analyzers with their resolution and type of metabolomics analysis. Quad—quadrupole; TOF—time of flight; Q-TOF—quadrupole-time of flight, FTICR–MS—Fourier transform ion cyclotron resonance mass spectrometry.

Plant metabolome is a complex mixture of low-molecular-weight compounds that vary in their diversity and abundance. Nuclear magnetic resonance (NMR) spectroscopy and mass spectrometry (MS) are the most commonly used analytical platforms to analyze the metabolome [179]. Compared to MS, NMR is non-destructive, non-selective, enables structural elucidation and cross-laboratory comparisons and is thus widely used for non-targeted metabolomics. However, the major disadvantage of NMR is its low sensitivity [180,181]. Although the major advantage of MS is its high sensitivity compared to NMR, the different mass analyzers used in these studies, which vary in their resolution, affect the outcome of the analysis (Figure 2; [182]).

MS is coupled with liquid chromatography (LC) or gas chromatography (GC) to separate the compounds in a complex mixture. GC–MS is most effective for volatile compounds and its efficiency can be improved by derivatizing the samples to increase the volatility and stability of compounds. The high polarity compounds can be well detected with LC–MS. Hence, GC–MS and LC–MS together provide a more comprehensive information about plant metabolome [183]. The disadvantages of MS based analysis include the presence of matrix effect, ion suppression that would lead to wide variation in signal intensities and selectivity for specific analytes [181]. Methods used for GC- or LC-based chromatographic separation would affect the ability to identity and quantitate the metabolites. None of the instruments or methods can entirely detect all the compounds in the plant sample and, they can vary in their strengths and limitations. [183]. The results of plant metabolome analysis in various studies may be partly attributed to different methods and instruments employed. Plant metabolome can also vary with plant age, development stage, environmental conditions and fertilizer regimes [58]. Another drawback in plant metabolomics is the lack of robust libraries for the identification of compounds [184].

## 6. Conclusions

Plant metabolites are pre-requisites to initiate and maintain the plant–AMF symbiotic association. Briefly, the plant–AMF association begins after recognizing specific metabolites by both plants and AMF. Further, the plant–AMF symbiotic association modifies the plant defense system starting from the signaling and carotenoid pathway, and expanding to the phenylpropanoid pathway. The reprogramming of plant secondary metabolism due to AMF symbiosis enables the plant to better tolerate abiotic and biotic stresses. The increase in abundance of the antioxidant compounds in the plant explains their enhanced resistance against pests and pathogens and the improved quality of plant products. Furthermore, the carbon demands of AMF influence the primary metabolism of the plant that affects the abundance of sugars, amino acids and, organic acids. Many of these primary metabolites also alleviate various abiotic stresses such as drought and salinity. Not only the point of contact of AMF, i.e., roots show the modification of plant metabolome, but the entire plant metabolome exhibit considerable variation with AMF association. Thus, the symbiotic association of plants with AMF modulates the plant metabolome to ensure the colonization of AMF and to obtain carbon benefits from the plants. The benefits of AMF symbiosis to plants may arise from a combination of increased plant biomass and reprogrammed plant primary and secondary metabolome. However, studies have also found alternate results in which AMF association makes the plant vulnerable against pathogens [185] and decreases the biomass of the plants [35]. The outcome of the plant–AMF symbiosis is highly species-specific, where some AMF associations result in a positive outcome, while others exhibit neutral or negative outcomes (parasitism; [35]). The ability of both plant species and AMF species to modulate the primary and secondary metabolites may partly contribute to this differential outcome in plant–AMF symbiosis. Hence, it is important to direct our efforts towards understanding the reprogramming of plant metabolome elicited by different AMF species when colonizing the same plant species or different plant species. The reprogramming of plant metabolome should also be captured at different stages of the symbiotic association and different developmental stages of plants to better understand the outcome of plant–AMF symbiosis. A comprehensive understanding of the AMF-mediated changes in plant metabolome would help in deciphering the mechanisms underlying plant tolerance to biotic and abiotic stresses and improving the product quality of crops. 

## Figures and Tables

**Table 2:** Summary of secondary metabolites modulated by AMF.

Secondary Metabolites	Plant Parts	AMF Species	Plant Species	Environmental Condition	Increase/Decrease	Reference
**Carotenoid pathway**						
Blumenols	Roots	*Rhizophagus irregularis*	*Senecio jacobea (Ragwort plant)*	-	Increase	[118]
Blumenols	Shoot	*Rhizophagus irregularis*	*Nicotiana attenuate*	-	Increase	[59]
Carotenoids	Leaves	*Glomus intraradices and G. mossae*	Lettuce	-	Increase	[120]
Cyclohexenone conjugates	Roots	*Glomus intraradices*	*Hordeum vulgare*	-	Increase	[121]
Cyclohexenone derivatives with blumenin	Roots	*Glomus intraradices*	*Hordeum vulgare and Triticum aestivum*	-	Increase	[122]
Cyclohexenone derivatives	Roots	*Glomus intraradices, Glomus mossae and Gigaspora rosea*	*Hordeum vulgare, Triticum aestivum and Zea mays*	-	Increase	[123]
	Roots	*Glomus intraradices*	*Tobacco and Tomato plants*	-	Increase	[87]
	Roots	*Glomus intraradices*	*Allium porrum*	-	Increase	[124]
	Roots	*Glomus intraradices*	*Medicago truncatula*	-	Increase	[70]
Mono-, di-, and branched triglycosides of blumenol	Roots	*Glomus intraradices*	*Ornithogalum umbellatum*	-	Increase	[125]
Mycorradicin	Roots	*Glomus intraradices*	*Ornithogalum umbellatum*	-	Increase	[125]
Sesquiterpenoid cyclohexenone derivatives	Roots	*Glomus intraradices*	61 members of poaceae	-	Increase	[126]
**Phenylpropanoid pathway**						
Bound phenolics	Roots	*Glomus intraradices*	*Medicago truncatula*	-	No change	[70]
Caffeoylshikimate	Leaves	*Rhizophagus irregularis*	*Salix purpurea*	-	Increase	[89]
Coniferyl alcohol	Roots	*Rhizophagus irregularis+ Funneliformis mossae*	*Solanum lycopersicum*	-	Increase	[64]
Coumarate	Leaves	*Rhizophagus irregularis*	*Salix purpurea*	-	Increase	[89]
Coumaryl acetate	Leaves	*Rhizophagus irregularis*	*Salix purpurea*	-	Increase	[89]
Coumaryl alcohol	Roots	*Rhizophagus irregularis+ Funneliformis mossae*	*Solanum lycopersicum*	-	Increase	[64]
Daidzein (Isoflavonoid)	Roots	*Glomus intraradices*	*Medicago truncatula*	-	Increase	[70]
Epicatechin	Leaves	*Rhizophagus irregularis*	*Salix purpurea*	-	Decrease	[89]
Ferulic acid	Roots	*Rhizophagus irregularis+ Funneliformis mossae*	*Solanum lycopersicum*	-	Increase	[64]
Flavonoids	Leaves	*Funneliformis mossae*	*Cucumis sativa (Cucumber)*	Chilling stress	Increase	[119]
Flavonoids	Leaves	*Gigaspora albida+ Acaulospora longula*	*Anadenanthera colubrina*	Increasing P concentration	Increase	[69]
Hydroxycinnamates	Shoots	*Rhizophagus irregularis*	*Cichorium intybus*	Metal toxicity	Increases without stress, no difference under stress	[127]
Hydroxycinnamic acid amides	Roots	*Glomus intraradices*	*Hordeum vulgare*	-	Increase	[128]
Hydroxycinnamic acid amides	Roots	*Glomus intraradices*	*Hordeum vulgare and Triticum aestivum*	-	Increase	[122]
Lignans: Secoisolariciresinol Yatein	Root	*Rhizophagus irregularis(Ri), Funneliformis mossae(Fm) and Claroideoglomu etunicatum(Ce)*	*Solanum lycopersicum*	Salt stress	Increase	[86]
Lignin	Leaves	*Funneliformis mossae*	*Cucumis sativa (Cucumber)*	Chilling stress	Increase	[119]
Luteolin-7-O-glucoside	Leaves	*Rhizophagus irregularis*	*Salix purpurea*	-	Increase	[89]
Malonylononin (Isoflavonoid)	Roots	*Glomus intraradices*	*Medicago truncatula*	-	Increase	[70]
Monolignans	Roots	*Rhizophagus irregularis+ Funneliformis mossae*	*Solanum lycopersicum*	-	Increase	[64]
Ononin (Isoflavonoid)	Roots	*Glomus intraradices*	*Medicago truncatula*	-	Increase	[70]
Phenolic acids	Roots+ shoots	*Glomus mossae*	Groundnut	-	Increase	[128]
Phenolic compounds: Cinnamic acid *p*-coumaric acid caffeic acid ferulic acid	Leaves	*Funneliformis mossae*	*Cucumis sativa (Cucumber)*	Chilling stress	Increase	[119]
Phenols	Leaves	*Gigaspora albida+ Acaulospora longula*	*Anadenanthera colubrina*	Increasing P concentration	Increase	[69]
Pinostrobin	Leaves	*Rhizophagus irregularis*	*Salix purpurea*	-	Increase	[89]
Quercetin (flavonoid)	Stems and leaves	*Mixture of Glomus fasciculatum, G. mossae, and G. intraradices*	*Vitis vinifera*	-	Increase	[129]
Rutin	Leaves	*Rhizophagus irregularis*	*Salix purpurea*	-	Increase	[89]
Scopoletin and its glucoside scopolin	Roots	*Glomus intraradices*	*Tobacco and Tomato plants*	-	Decrease	[87]
Scopolin	Leaves	*Rhizophagus irregularis*	*Salix purpurea*	-	Decrease	[89]
Tannins	Leaves	*Gigaspora albida+ Acaulospora longula*	*Anadenanthera colubrina*	Increasing P concentration	Increase	[69]
*Alkaloids*						
Benzylisoquinoline alkaloids	Roots	*Rhizophagus irregularis+ Funneliformis mossae*	*Solanum lycopersicum*	-	Increase	[64]
Pyrrolizidine alkaloids	Roots	*Rhizophagus irregularis*	*Senecio jacobea (Ragwort plant)*	-	Increase	[118]
Trigonelline (pyridine alkaloid)	Roots	Gigaspora rosea	*Prosopis laevigata*	-	Increase	[130]
*Phytoalexins*						
Rishitin	Roots	*Glomus etunicatum*	Potato plantlets	*Rhizoctonia solani* pathogen	Increase	[131]
Solavetivone	Roots	*Glomus etunicatum*	Potato plantlets	*Rhizoctonia solani* pathogen	Increase	[131]
*Antioxidants*						
Bioactive compounds: Picrocrocin Crocin II Quercitrin	saffron	*Rhizophagus intraradices*	*Crocus sativus* (saffron)	-	Increase	[132]
Caffeic acids	Shoot	*Glomus caledonium and Glomus mossae*	Sweet basil	-	Increase	[133]
Caffeic acids	Shoot	*Glomus intraradices*	Sweet basil	-	Same as non-mycorrhizal	[133]
Rosmarinic acid	Shoot	*Glomus caledonium and Glomus mossae*	Sweet basil	-	Increase	[133]
Rosmarinic acid	Shoot	*Glomus intraradices*	Sweet basil	-	Same as non-mycorrhizal	[133]
**Plant defense and hormones**						
Abscisic acid	Leaf	*Rhizophagus irregularis*	Medicago truncatula	-	Increase	[134]
Catalpol	Leaf	*Rhizophagus irregularis*	** Monocot and dicots	-	Increased in *Plantago lanceolate*, slightly decreased in *Veronica chamaedrys*	[76]
Glucosinolates	Root	*Funneliformis mossae*	*Triticum aestivum and Triticum durum*	Water stress	Mostly decreased	[92]
Jasmonic acid Methyl jasmonate	Root	*Rhizophagus irregularis(Ri), Funneliformis mossae(Fm) and Claroideoglomu etunicatum(Ce)*	*Solanum lycopersicum*	Salt stress	Increase	[86]

** Monocot and dicots: *Plantago lanceolata*, *Plantago major*, *Veronica chamaedrys*, *Medicago truncatula* (Dicots) and *Poa annua* (Monocot).
